# Hyperactive browning and hypermetabolism: potentially dangerous element in critical illness

**DOI:** 10.3389/fendo.2024.1484524

**Published:** 2024-11-21

**Authors:** Lu Huang, Lili Zhu, Zhenxiong Zhao, Shenglu Jiang

**Affiliations:** ^1^ Department of Basic Medical Sciences, Taizhou Central Hospital (Taizhou University Hospital), Taizhou, China; ^2^ Department of Plastic and Reconstructive Surgery, Taizhou Enze Hospital, Taizhou, China

**Keywords:** brown adipose tissue, beige adipose tissue, critical illness, thermogenesis, hypermetabolism, metabolic disorder

## Abstract

Brown/beige adipose tissue has attracted much attention in previous studies because it can improve metabolism and combat obesity through non-shivering thermogenesis. However, recent studies have also indicated that especially in critical illness, overactivated brown adipose tissue or extensive browning of white adipose tissue may bring damage to individuals mainly by exacerbating hypermetabolism. In this review, the phenomenon of fat browning in critical illness will be discussed, along with the potential harm, possible regulatory mechanism and corresponding clinical treatment options of the induction of fat browning. The current research on fat browning in critical illness will offer more comprehensive understanding of its biological characteristics, and inspire researchers to develop new complementary treatments for the hypermetabolic state that occurs in critically ill patients.

## Introduction

Critical illness, aroused by severe accidental or iatrogenic trauma, widespread invasion of highly virulent pathogens, aggressive cancer progression, etc., is mainly characterized by potentially fatal changes in the hypothalamic-pituitary-axis, which therefore usually demonstrates a desperate need for organ support and close monitoring but still inevitably produces a relatively high proportion of deaths (1, 2). Hypermetabolism accompanied by severe systemic impairment was defined by a significant increase in measured resting energy expenditure relative to predicted resting energy expenditure (3). And the more specific features of hypermetabolism in critical illness such as extensive thermal injury, cancer cachexia and sepsis are represented by accelerated overall energy expenditure and persistent widespread metabolic imbalance, including the excessive accumulation of free fatty acids (FFAs), amino acids and glucose in circulation (4–6). The significant elevations in resting energy expenditure resulted in the uncontrolled wasting of human nutritional components, and the subsequent systemic disorders of metabolism, endocrinology, inflammation and immunity followed the prolonged catabolic reactions ultimately (7, 8). Patients with critical illness are always experiencing severe disturbance in life support systems and in desperate need for nutritional supply and proactive intervention for homeostasis maintenance (9). Regretfully, hypermetabolic conditions further aggravate the imbalance of multiple important systems and increase the overall mortality rate in patients with critical illness (10).

Beige adipose tissue (Beige AT) was observed in traditional white adipose tissue (WAT) depots under specific stimulus with acquired brown adipose tissue (BAT) characteristics, mainly morphologically showing the sparsely distributed small adipocytes enrich in multilocular lipid droplets and mitochondria among large adipocytes, accompanied by more substantial vascularization and innervation histologically (11). Besides, activated uncoupling thermogenesis of the induced beige adipocytes mediated by the overexpressed uncoupling protein 1(UCP1) facilitates the rapid exhaustion of energy-containing multi-carbon units, which corresponds to the efficient specific uptake of circulating substrates for lipid and glucose metabolism (12–14). During the acclimation process to cold exposure, as firstly reported decades ago to be the traditional well-acknowledged browning stimulus, the enhanced thermogenesis of induced beige adipose tissue improves the individual’s ability to withstand low temperature (15, 16).

Due to its energy dissipation feature, browning of WAT was also regarded as the potential target for the therapy of obesity and metabolic syndrome, which therefore promoted the vigorous development of browning-related agonists (17, 18). For all types of populations suffering from obesity, especially those who lack the ability to actively exercise, promoting browning of adipose tissue can simultaneously eliminate subcutaneous and visceral fat through non-shivering thermogenesis and improve obesity phenotype (19). In addition, adipose tissue browning can elevate basal metabolic levels and correct metabolic disorders including impaired lipid metabolism and glucose metabolism to a certain extent. Therefore, for metabolic syndrome patients with symptoms such as hyperglycemia, hyperlipidemia and insulin resistance, browning of adipose tissue is also a potential therapeutic strategy to promote their overall metabolism (20). Recent research has discovered another interesting phenomenon, that is, in addition to participating in shivering thermogenesis, skeletal muscles can also undergo non-shivering thermogenesis under the stimulation of various browning stimuli, and may work synergistically with brown or beige adipose tissue through internal mechanisms such as paracrine effects (21–24). Therefore, when extensive fat browning occurs, we should also explore the possible simultaneous changes in skeletal muscle and carry out targeted intervention in subsequent studies, especially in the field of metabolic disorder.

However, recent researches discovered that WAT browning was observed also in the progression of severe burns, sepsis and cancer cachexia (25–27). Although previous studies, especially the adipocyte subpopulations located in different fat depots identified based on single-cell sequencing, have different browning potentials, under the strong browning stimulation in the context of critical illness, the adipose tissue in both subcutaneous and visceral fat depots is basically activated (28, 29). Regretfully, the existing evidence suggested that extensive browning of WAT further exacerbated hypermetabolism in critical illness (30). Patients in critical illness, especially skinny individuals, are already under extreme consumption of negative nitrogen balance, which could be further aggravated with totally disturbed metabolic homeostasis after the induction of active beige adipose tissue (31). This phenomenon to some degree clarified the obesity paradox, which indicated the slightly obese individuals possessed longer survival than the slim or normal weight ones as shown by large-scale demographic analysis (32, 33). In this review, we will demonstrate the adaptive changes and specific behavior of adipose tissue under metabolic stresses, especially the browning of WAT, and discuss the possible mechanism of massive beige adipose tissue emergence, medical risk to multiple systems as well as its potential prognostic and therapeutic values with the aim for more comprehensive and efficient intervention of patients with critical illness.

## Browning of WAT in critical illness

Browning of traditional white adipose tissue, especially subcutaneous WAT, was first observed in mice exposed to cold stress and manifested as sporadic clusters of small mitochondria-rich cells similar to brown adipocytes distributed in the adipose tissue (34). The induced beige adipocytes tended to rapidly release heat energy by consuming substrates such as free fatty acids in circulation through UCP1-mediated uncoupling thermogenesis, which apparently enhanced the ability of cold endurance for the mice (35). Subsequent studies confirmed that browning of WAT can be triggered by *in-vivo* application of β3 adrenoreceptor agonists, peroxisome proliferator-activated receptor (Pparγ) agonists, dietary intake of natural ingredients such as capsaicin or even adequate exercises (36–39). With regards to humans, there is considerable amount of literature concerning the browning of both subcutaneous and visceral WAT under similar stimulus with sufficient evidence acquired from the discoveries of anatomy and imaging examination especially PET-CT. However, recent studies depicted that significant systemic stress in critical illness, such as burns, severe trauma, sepsis and cancer cachexia, was always accompanied by extensive browning of white adipose tissue.

In burn cases, surgeons and researchers are most concerned about the extensive liquefaction and necrosis of adipose tissue, especially the subcutaneous white adipose tissue adjacent to the burn sites, which can lead to further inflammatory cascades, catastrophic infection, and a series of other complications (40). However, in 1991, Rothwell et al. firstly reported that brown adipose tissue in rats was also involved in the stress response after scalding, which was manifested in the suppression of weight gain and the increase in individual metabolic levels accompanied by the activation of BAT function (41). In the past decade, pathophysiological research on adipose tissue confirmed that it plays a substantial endocrine and paracrine role in many diseases correlated with metabolic disorders (42). Therefore, some scholars began to explore the relationship between adipose tissue and the hypermetabolic state in the context of burns (43). Zhang et al. discovered the increased Ucp1 mRNA expression in mice brown adipose tissue after burn injury in 2008, and moreover, the resected subcutaneous white adipose tissue also showed increased Ucp1 expression (44). This finding was noteworthy because it indicated that after burns, not only the brown adipose tissue located in the fixed anatomical areas increases its non-shivering thermogenesis, but the subcutaneous white adipose tissue also undergoes extensive browning, and the two together intensify individual’s hypermetabolism. Later studies carried out by Sidossis et al. indicated that the similar phenomenon was also observed in humans after severe burns, and that the significant morphological and functional changes of resected subcutaneous WAT, such as the wide distribution of multilocular adipocytes, increase of uncoupling protein 1 (UCP1), increase of mitochondrial density and respiratory capacity, were enough to prove the occurrence of browning (45).

In cancer, traditional white adipose tissue is always considered an accomplice because obesity is associated with both increased cancer incidence and progression in multiple tumor types (46, 47). Therefore, previous studies tended to focus on the remodeling of adipose tissue microenvironment, including inflammation, vascularity and fibrosis, which were believed to directly influence tumor initiation and progression (48). However, Wagner et al. pointed out that another phenotypical switch from WAT to BAT was obvious in mice with cancer-associated cachexia (49). Later clinical studies accomplished by Bredella et al. performed long-term follow-up of tumor patients and utilized PET/CT scanning to detect BAT activity in patients with different tumor prognosis (50, 51). It was found that BAT is significantly activated during tumor progression and can even be used as a marker of tumor recurrence or metastasis to a certain extent.

Besides, browning of WAT was also observed in nonobese mice after the induction of polymicrobial sepsis, which was absent in obese mice with less release of norepinephrine. Other disorders that cause excessive adrenergic stress, such as hyperthyroidism and extensive wounds, also have pathological basis for inducing browning of WAT in both model animals or humans as reported previously (52–54).

## Hazards of WAT browning in critical illness

As a famous *in vivo* thermal power plant that continuously utilizes circulating glucose or lipids as fuel, there is no denying the fact that brown or beige adipose tissue could help to preserve the physiological range of body temperature in severe burns or extensive wounds with obviously damaged soft tissue barrier (55–57). And as part of the body’s stress response, browning of WAT can promote decomposition of lipid droplets and release of substrates into the blood, thereby increasing the basic energy supply of target cells throughout the body in a short period of time (58). In the meantime, beige adipose tissue or activated brown adipose tissue can absorb glucose or lipids from circulation, improving insulin resistance and hyperlipidemia (59). On the other hand, browning of adipose tissue was also related with type II inflammation pattern, which might help reduce the production of mediators of oxidative stress and immune responses and protect the individuals from the catastrophic inflammatory storm (60, 61). Interestingly, recent study in cancer revealed that the ability to compete for energy substrates in activated BAT after cold stimulation help starve the cancer cells, which eventually prolonged patient survival time (62). This study also illustrated the dominant position of brown or beige adipose tissue in competing for metabolic substrates, even in direct competition with tumor cells.

**Figure 1:**
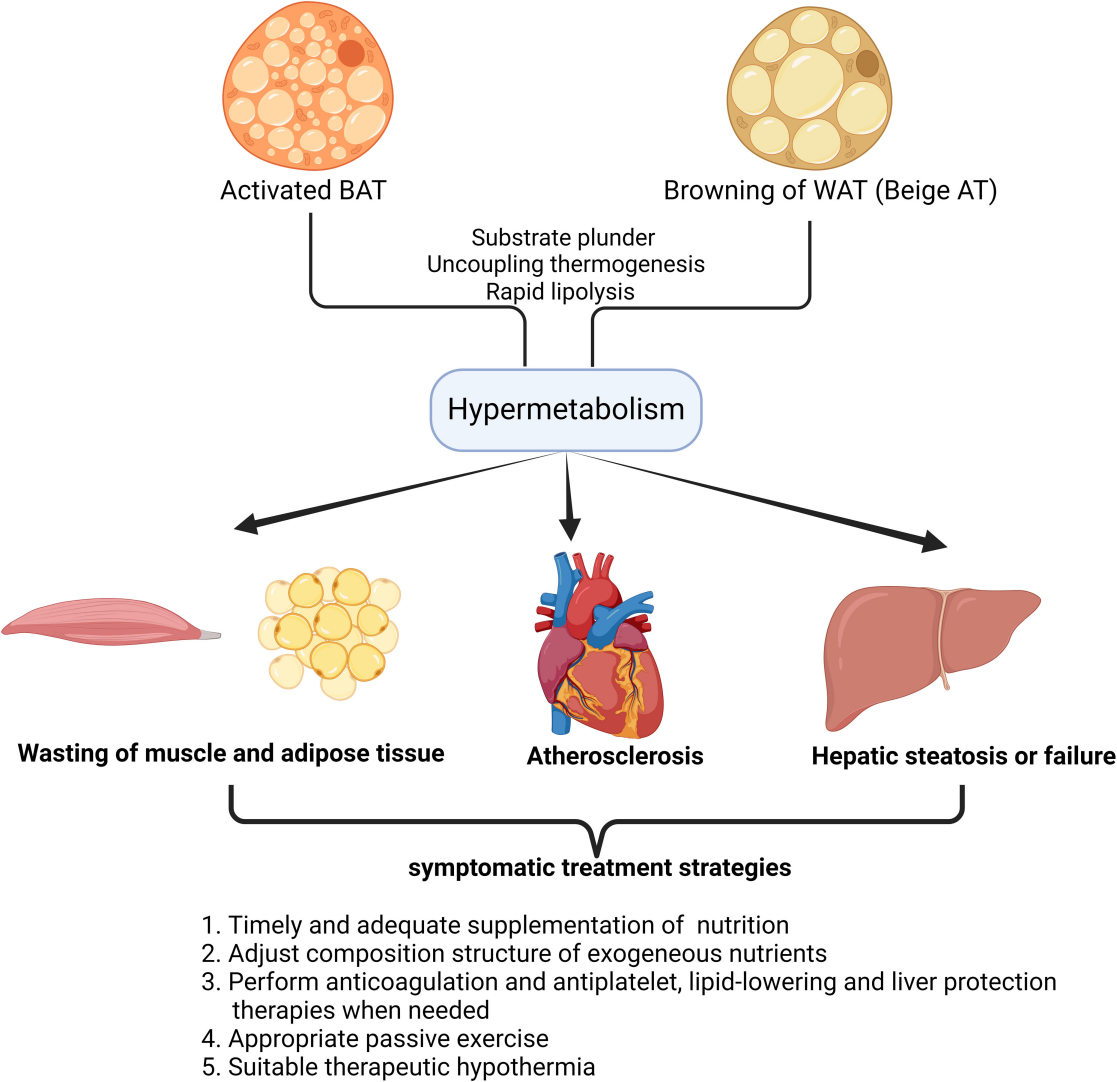
Damage patterns of target organs caused by hypermetabolism with enhanced fat browning in critical illness and symptomatic treatment strategies. The schematic diagram illustrates the impact of fat browning(Activated BAT or Beige AT) on target organs in humans. A number of organs and tissues have been found to get impaired in hypermetabolism, as a result of fat browning in critical illness. This simplified overview also delineates the alternative symptomatic treatment strategies based on the understanding of the pathological process in target organs after the occurrence of hypermetabolism in critical illness.

However, there are also serious systemic risks in critical illness as reported recently (**Figure 1**). Firstly, hypermetabolism in critical illness is quite normal with excessive energy consumption and elevated resting energy expenditure (REE) (63). However, previous studies indicated the activated BAT and beige adipose tissue can exacerbate the body’s hypermetabolic state (64). Even though the hypermetabolic state in critical illness at first mobilizes the defense functions and prepares the body for further harms, its long-term maintenance does cause unnecessary waste of nutrients, rapid exhaustion of exogenously supplemented energy and bring systemic disorders of glucose and lipid metabolism, nitrogen balance, cardiovascular homeostasis, immunity, etc (65). In decompensated state of critical illness, hypermetabolism can directly induce the dysfunction of multiple organs.

Besides, in hypermetabolism, it should never be neglected that adipose tissue and muscles are continuously wasting after the overactivation of BAT or beige AT (66). The accumulation of adipose tissue has always been regarded as the culprit of many metabolic-related diseases, and keeping a high body mass index is considered a disease state (67). However, excess adipose tissue provides fuel reserves to bridge the gap between decreased intake and elevated requirements in critical illness (68). Therefore, excessive mobilization of energy stored as triglycerides in adipose tissue especially after the browning of both subcutaneous and visceral WAT reduces the body’s ability to respond to more damaging internal and external factors (69). Furthermore, previous studies indicated that muscle wasting also inevitably accelerates after the browning of WAT in cachexia (70). As a typical feature and upstream modulator of beige adipose tissue, enhanced lipolysis can be observed in burns or cancer-related cachexia, which is characterized by severe weight loss, systemic inflammation, muscle and adipose tissue wasting (71). Das et al. demonstrated that attenuating lipolysis via genetic ablation of adipose triglyceride lipase preserves both the adipose tissue and muscle (72). Wasting of both adipose tissue and muscle after the overactivation of BAT and beige AT symbolizes the uncontrollable dissipation of substrate depots, therefore lack of nutrition supplement in lean individuals can be devastating (73).

In addition, long-term release of lipids into circulation after WAT browning causes the imbalance of plasma lipid profiles and accelerates the progression of atherosclerosis, hepatic steatosis as well as immune suppression (74, 75). It is notable that even in lean individuals, the characteristic pathological changes caused by lipid metabolism disorders are still obvious.

Considering that both brown and beige fat can be activated in critical illness, the detailed relationship as well as the difference between brown adipose tissue and beige adipose tissue should also be explored in the future. Nowadays, it’s clear that in critical illness, BAT itself increases the production efficiency as a thermal energy factory, presenting with enhanced uptake of substrates and uncoupling thermogenesis. At the same time, BAT can release a variety of batokines through endocrine to affect multiple target organs, including white adipose tissue, whose lipolysis can be obviously initiated (76). As for beige adipose tissue, first of all, its browning is accompanied by significant lipolysis and the removed lipid droplets can’t be completely consumed by themselves (77). Therefore, a large amount of lipids are released into the blood during the process of browning, and some of them are absorbed by BAT as fuel. But the excessive accumulation of lipids in blood can directly disturb the metabolism, causing atherosclerosis, hepatic steatosis and etc (78). Therefore, unlike brown adipose tissue which acts as a thermal factory and signal tower, beige adipose tissue is more like an energy reserve warehouse and a temporary heat-generating factory during wartime. Unfortunately, the overuse of both tends to exert a negative effect in the heat of battlefield (79).

## Possible mechanisms of WAT browning in critical illness

**Figure 2:**
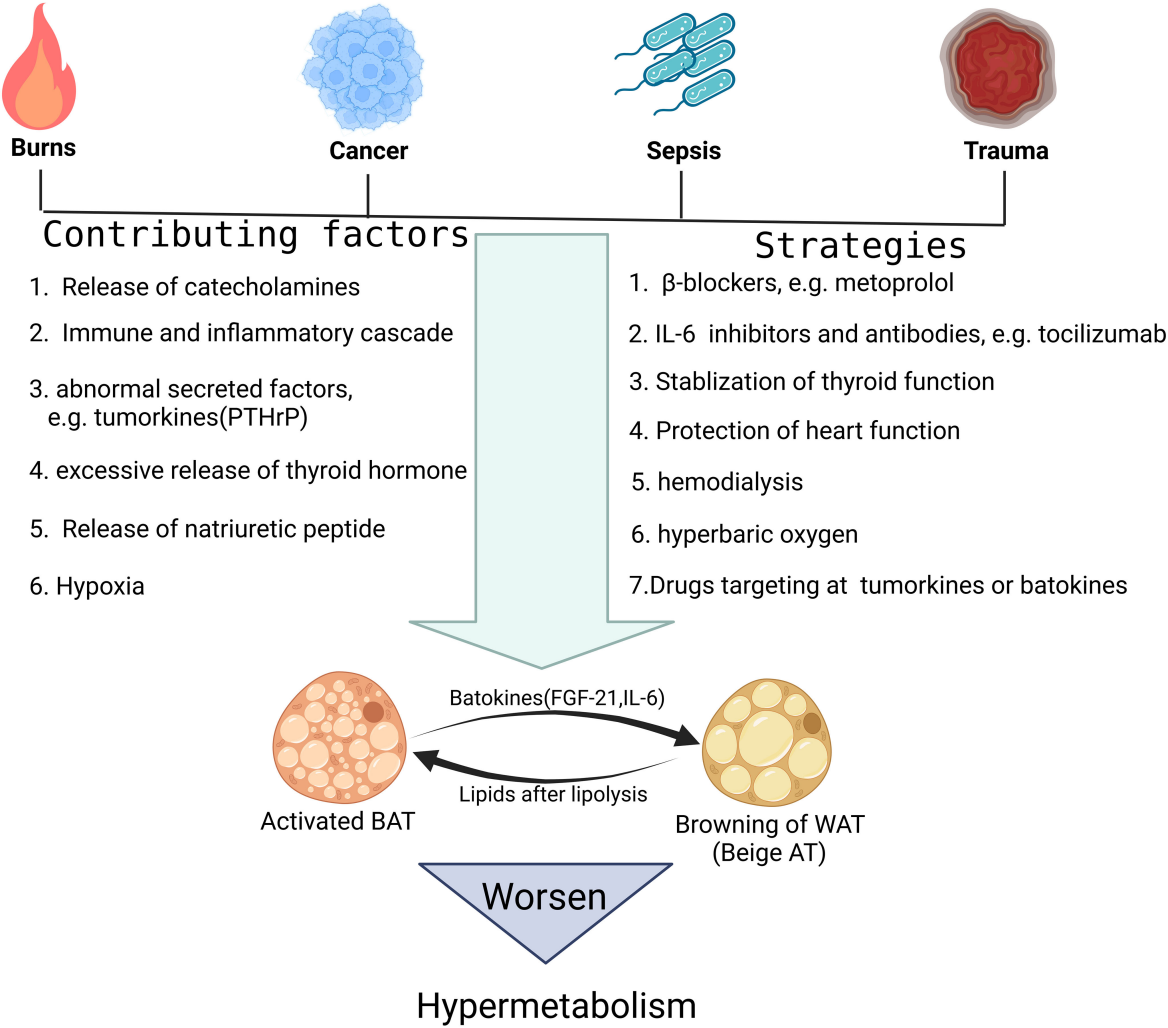
Possible mechanisms causing fat browning in critical illness and corresponding targeted treatment strategies. This figure lists the representative pathological damage factors that tend to activate fat browning. In addition, it is also shown that several molecular pathways, such as the β-adrenergic pathway and IL-6 mediated inflammatory pathway, can trigger the fat browning process in critical illness. At last, the specific treatment options aiming at interfering with these molecular mechanisms and targets are discussed.

Referring to the activation mechanism of WAT in response to traditional browning stimuli such as cold, upregulation of adrenergic pathway is of crucial importance, and the sympathetic nervous system is always overactivated in critical illness (**Figure 2**) (80). In burns, previous clinical research indicated that hypercatecholamines accompanied by excessive activation of the sympathetic nervous system not only appear immediately after trauma, but can also be maintained for a long time (81). Further basic research confirmed that this sustained hypercatecholamines promoted activation of BAT and browning of WAT, which together contributed to the formation of hypermetabolism in critical illness (82, 83). However, recent studies suggested M2 macrophages can also release catecholamines locally and directly induce the activation of β3-AR pathway of adipocytes. This mechanism relies on type II inflammatory response, in which the upstream molecule that induces macrophages to polarize toward M2 type might be IL-4, since mice lacking IL-4 signaling exhibited impairments in browning. Interestingly, in obese patients, white adipose tissue always experiences a chronic inflammation response characterized by M1 macrophage infiltration, so this mechanism partially explains the lower degree of browning in obese individuals in critical illness and also adds further understanding to obesity paradox (84, 85). Besides, other mediators that regulate polarization of macrophages were studied recently in burn patients and among them, palmitate drew much attention since it is one of the free fatty acids(FFAs) secreted by adipocytes undergoing lipolysis and is abundant in serum after burns (86). This positive feedback mechanism continuously strengthens the upstream browning stimulation, leading to further lipolysis and browning of WAT.

In addition to the secretion of catecholamines, other endocrine factors and cytokines have also been implied to be associated with browning of WAT in critical illness. IL-6, for example, has been validated to affect the browning process of WAT because mice lacking the IL-6 gene have impaired WAT browning in response to burns and cancer, and that the enhanced activation of IL-6 signaling induces WAT browning and increases energy expenditure in patients with critical illness (87, 88). As a classic cytokine, IL-6 can be secreted by a variety of cells, such as various immune cells, fibroblasts, etc. during infection and trauma, and it has a wide range of regulatory effects on many target cells (89). However, recent studies in critical illness revealed its underlying mechanism also involves macrophage polarization toward M2 type and theoretically, clearance of the infiltrated macrophages in adipose tissue can counteract the browning-promoting effect of IL-6 (90).

Batokines, defined as the BAT-derived regulatory molecules that act in a paracrine or autocrine manner, are believed to influence systemic metabolism and convey the metabolic effects of BAT activation to other tissues and organs. Interestingly, in many cases, such as under cold stimulation, the activation of BAT is often accompanied by browning of WAT, which suggests that WAT may also be one of the target tissues acted upon by part of the batokines (91). Previous studies firstly identified fibroblast growth factor 21(FGF-21), IL-6 and neuregulin 4 as the BAT-derived endocrine factors, or batokines (92). As mentioned above, IL-6 is an important signaling molecule that causes extensive browning of WAT in severe cases such as burns (93). FGF-21, although mainly produced by the liver and involved in hepatic lipid and carbohydrate metabolism, it can also be produced by activated BAT, inducing browning of WAT by binding to FGF-21 receptors and β-klotho co-receptors on adipocytes and activating the p38 MAPK pathway (94–96). Later studies have confirmed that the persistent type I inflammatory response in obese patients can interfere with the expression of β-klotho co-receptors through the production of TNF-α, thereby inhibiting the function of FGF-21 (97). While the anti-inflammatory microenvironment dominated by M2 macrophages in tumor patients relieves this inhibition pattern and promotes the efficient conduction of FGF-21 signaling and the rapid browning of WAT (98). Therefore, the batokine profile should be further investigated, considering the exact signaling link between BAT and WAT in critical illness.

Also in tumor patients, as cell clusters with obvious heterogeneity, tumors also have their own specific secretion profile, or tumorkines, some of which may affect the browning process of WAT (99, 100). For example, parathyroid hormone related protein (PTH-rP), which can be released by tumors into circulation, has a broad range of target organs, including skin, cartilage, placenta, bone and adipose tissue as well (101). In specific, under the stimulation of PTH-rP, browning program can be initiated in WAT of both humans and rodents, which aggravates hypermetabolism in cancer cachexia (102).

Considering that thyroid hormone, mainly triiodothyronine(T3), can increase the activity of UCP1 distal enhancer, which contains a binding site for the T3/TRβ complex, by activating thyroid hormone receptors on adipocytes, the role of thyroid hormone in hypermetabolism cannot be ignored (103). After all, critical illness is often accompanied by excessive release of thyroid stimulating hormone (TSH) and thyroid hormone (104). Previous studies also clarified that T3 is mainly released by the thyroid gland, but adipocytes themselves can catalyze thyroxine(T4) into T3 through type II iodothyronine 5’-deiodinase(DIO2) (105). Therefore, thyroid hormone, including T3 and T4, can both cause browning of WAT and should be closely monitored in critical illness. In addition, thyroid hormone can also increase the metabolic activity of many target cells, which acts synergistically with activated brown or beige adipose tissue, jointly exacerbating the hypermetabolic state (106).

In the same way, patients with critical illness are often accompanied by abnormalities in cardiac function, such as heart failure (107). As indicator molecules characteristically secreted by the ventricle in heart failure, the cardiac natriuretic peptide family, consists of three members: atrial natriuretic peptide, brain natriuretic peptide (BNP) and C-type natriuretic peptide (108). Among them, atrial natriuretic peptide and BNP achieve their biological effects through natriuretic peptide receptor A, while C-type natriuretic peptide binds to guanylyl cyclase receptor. In WAT of patients with critical illness, natriuretic peptide (NP) signaling activates cyclic guanosine monophosphate protein kinase (cAMP), and then phosphorylates hormone-sensitive lipase (HSL) and perilipin, breaking down lipid droplets and providing raw materials for mitochondrial respiration (109). This mechanism mediated by the cardiac natriuretic peptides complements the theoretical framework and elucidates more intrinsic causes of the browning of WAT especially in patients with cardiac dysfunction.

At last, in some research, hypoxia is considered to be another contributing factor of WAT browning (110). In critical illness such as sepsis, tissue hypoxia is very common and can become more severe as the condition worsens (111). Our previous research on adipose tissue transplantation *in vivo* and anaerobic culture of white adipocytes *in vitro* indicated the appearance of multiocular, mitochondria-rich, small-volume brown fat-like adipocytes under hypoxia (112). Cao et al. reported that the upregulation of hypoxia induced factor(HIF) and vascular endothelial growth factor(VEGF) enhances microvascularization in adipose tissue and also activates the sympathetic nervous system by promoting the release of norepinephrine, which together contribute to the browning of WAT under hypoxia (113).

## Potential therapeutic significance of WAT browning in critical illness

Hypermetabolism in critical illness is undoubtedly harmful. On the one hand, it causes excessive energy depletion of the body; while on the other hand, it also aggravates the individual’s metabolic disorder and destroys the homeostasis of the whole body (114). Therefore, in critical illness, besides conventional treatment, the occurrence of hypermetabolic states should be noted and the corresponding treatment aiming at alleviating hypermetabolism should be adopted. In general, the hypermetabolic state in critical illness should be mainly dealt with in two aspects. One is to use various symptomatic treatment strategies to reduce the harmful effects on the body, and the other is to carry out targeted intervention for the cause of WAT browning (115).

In the context of the hypermetabolic state of critical illness, the first thing to bear the brunt is the rapid depletion of various substrates, including lipids, carbohydrates, amino acids, etc(**Figure 1**) (116, 117). Timely and adequate supplementation of exogenous nutrition is naturally essential, but for a patient in hypermetabolic state, the lack of a relatively objective quantitative standard makes it very difficult to adjust nutritional support strategies (118). Calculating the basal metabolic rate (BMR) of a bedridden patient through indirect calorimetry may be a feasible solution, but currently there is a lack of a simple and efficient testing device in clinical practice (119). The question of whether the current amount of exogenous nutritional supplements in critical illness needs to be increased still needs further exploration (120). In addition, the composition structure of exogeneous nutrients also needs to be adjusted according to the patient’s specific metabolic state, especially the content of each metabolite in the circulation, so as to actively correct the metabolic disorder in patients with critical illness (121). At the same time, considering that hyperlipidemia in hypermetabolism is often accompanied by blood hypercoagulability and can further induce complications such as anticoagulation and liver cirrhosis, corresponding treatments such as anticoagulation and antiplatelet, lipid-lowering and liver protection should be applied simultaneously (122). For critically ill patients who are bedridden, muscles are consumed at an accelerated rate in hypermetabolism, and muscle fibers atrophy rapidly due to loss of use (123). Therefore, appropriate passive exercise and protein supplement cannot be ignored when formulating a comprehensive treatment plan. At last, we need to discuss the use of therapeutic hypothermia in critical illness. Although lowering the ambient temperature can alleviate the patient’s stress response and reduce some unnecessary energy consumption, too low temperature can obviously activate adipose tissue, induce non-shivering thermogenesis and even induce muscle trembling (124). Therefore, it is necessary to find a suitable low ambient temperature that cannot directly induce adipose tissue browning or muscle trembling.

Previous studies have well demonstrated that activated BAT or browning of WAT aggravates hypermetabolism, so treatment options targeting BAT or beige adipose tissue have significant clinical significance (**Figure 2**). Since the release of catecholamines caused by activation of the sympathetic nervous system can directly activate browning of adipose tissue, reducing the excitability of the sympathetic nervous system or the concentration of catecholamines is a feasible therapeutic strategy, such as using β-blockers (125). Coincidentally, the release of the thyroid hormones is also associated with activation of the sympathetic nervous system, so β-blockers, such as metoprolol, may also alleviate hypermetabolism by stabilizing thyroid function (126). Of course, the above discussion also proves that monitoring the concentration of catecholamines or thyroid hormones in the circulation also has certain reference value for adjusting treatment strategies. IL-6 or its receptor inhibitors and monoclonal antibodies are among the drugs used to inhibit inflammation in clinical practice (127). Some of these products have been widely used, such as the IL-6 receptor inhibitor tocilizumab (128). Here, we have clarified from previous studies that IL-6 mediated adipose tissue browning is involved in the formation of hypermetabolism in critical illness. Therefore, the use of IL-6 or its receptor inhibitors and monoclonal antibodies can theoretically correct hypermetabolism by reducing the degree of adipose tissue browning (88). When necessary, hemodialysis to remove excess circulating factors, such as IL-6, is also an option. Given that batokines, such as FGF21, and tumorkines, such as PTH-rP, are involved in hypermetabolism in critical illness, subsequently developed drugs targeting these molecules have significant potential in treating hypermetabolism. In addition, cardiac dysfunction or thyroid dysfunction in critical illness can further aggravate fat browning and hypermetabolism through the secretion of T3 and natriuretic peptides. Therefore, the clinical significance of protecting thyroid and heart function is further highlighted. Finally, considering the role of tissue hypoxia in promoting adipose tissue browning, fully improving tissue hypoxia through hyperbaric oxygen and other alternative treatments may also be an excellent option to correct hypermetabolism in critical illness.

## Conclusion

Hypermetabolism in critical illness brings significant risks to patients, while activation of BAT and browning of WAT are presumed to be an important cause of hypermetabolism as the previous studies suggest. Release of catecholamines caused by activation of the sympathetic nervous system, batokines such as IL-6, FGF-21 and tumorkines such as PTH-rP, as well as other neuroendocrine factors including thyroid hormone and cardiac natriuretic peptide, are the upstream modulators mediating adipose tissue browning in critical illness. This review highlights the role of activated BAT or WAT undergoing browning process in hypermetabolism of critical illness, and points out potential clinical solutions to correct hypermetabolism through the combination of symptomatic treatment and brown fat-specific treatment.
